# Challenges faced by health workers in providing counselling services to HIV-positive children in Uganda: a descriptive study

**DOI:** 10.1186/1758-2652-13-9

**Published:** 2010-03-07

**Authors:** Joseph Rujumba, Cissy L Mbasaalaki-Mwaka, Grace Ndeezi

**Affiliations:** 1 Department of Paediatrics and Child Health, College of Health Sciences, Makerere University, PO Box 7072, Kampala, Uganda

## Abstract

**Background:**

The delivery of HIV counselling and testing services for children remains an uphill task for many health workers in HIV-endemic countries, including Uganda. We conducted a descriptive study to explore the challenges of providing HIV counselling and testing services to children in Uganda.

**Methods:**

A descriptive study was conducted in the districts of Kampala and Kabarole in Uganda. The data were collected using semi-structured individual interviews and focus group discussions with health workers who are involved in the care of HIV-positive children. Key informant interviews were conducted with the administrators of the 10 study healthcare institutions. Quantitative data were summarized using frequency tables, while qualitative data were analyzed using the content thematic approach.

**Results:**

Counselling children was reported to be a difficult exercise due to some children being unable to express themselves, being dependent on adults for their care, being fearful, and requiring more time to open up during counselling. This was compounded by some caretakers' unwillingness and difficulty to disclose the HIV status of their children. Other issues about the caretakers were: lack of consistency in caretakers; old age; sickness; and poverty. Health workers mentioned the following as some of the challenges they face in the delivery of HIV counselling and testing services for children: lack of counselling skills; failure to cope with the knowledge demand; difficulty to facilitate disclosure; heavy work load; and lack of other support services. Institutions were found to be constrained by limited space and lack of antiretrovirals for children.

**Conclusions:**

The major challenges in the delivery of paediatric HIV services were related to the knowledge gap in paediatric HIV and the lack of counselling skills, as well as health system-related constraints. There is a need to train health workers in child-counselling skills, especially in the issues of disclosure, sexuality and sexual abuse, as well as in addressing fears related to death and an uncertain future, in order to improve paediatric HIV care. Provision of child-friendly services, guidelines and antiretroviral formulations for children may provide a window of hope to improve HIV counselling and testing services for children.

## Background

HIV/AIDS has had a devastating impact on both adults and children. Globally, more than 2.3 million children are estimated to be living with HIV/AIDS. Almost 90% of these children live in sub-Saharan Africa [1]. Recent estimates by the Joint United Nations Programme on HIV/AIDS (UNAIDS) indicate that about 130,000 children aged 0 to 14 years are living with HIV in Uganda [2].

International and national efforts to provide care and support for children who are infected and/or affected by HIV/AIDS, including provision of paediatric HIV treatment, are increasing. The "Unite for Children, Unite against AIDS" initiative by UNICEF/UNAIDS targets provision of either antiretroviral treatment or contrimoxazole, or both, to 80% of children in need [3]. However, the number of HIV-positive children under 15 years of age receiving antiretroviral therapy (ART) remains low [4-7]. Only 13% of the children in need of ART in sub-Saharan Africa receive it [8].

This could be a manifestation of the limited care and support services for HIV-infected children, including HIV counselling and testing services as an entry point for such care. In order to bridge the gap, the Uganda Ministry of Health and partner agencies are scaling up HIV counselling and testing services in the country as part of the ART and prevention of mother to child transmission (PMTCT) programmes.

Until recently, most of the HIV counselling and testing services in Uganda targeted adults. Currently, HIV counselling and testing services for children are available at the national teaching hospitals, regional hospitals and district hospitals. There are also some private, not-for-profit hospitals and non-governmental organizations, like The AIDS Support Organization, AIDS Information Centre, Joint Clinical Research Centre, Baylor College of Medicine Children's Foundation Uganda and the Mildmay Centre, which provide HIV care, including child counselling and testing.

In addition, the Uganda National Policy Guidelines for HIV counselling and testing provide for HIV counselling and testing of children aged 12 years and above without the knowledge or consent of parents or guardians, provided the children have the capacity to understand the implications of the test results [9]. For children who are below the age of 12 years, consent of the parent or guardian must be sought and documented. In the absence of a parent or guardian, the head of an institution can give consent on behalf of the child [9]. The policy further emphasizes the need for healthcare providers to counsel both the child and his/her parents or guardians [9].

Despite these advances, provision of HIV counselling and testing services for children has remained a difficult task for many health workers. Allen and Marshall note that the concerns of vulnerable populations, including children living with HIV, are often difficult and demanding for the patients, their families and the health workers [10]. With this background, we conducted a descriptive study to explore the challenges that healthcare workers face in the delivery of HIV counselling and testing services to children and their caretakers in Uganda.

## Methods

### Design, study sites, and participants

We conducted the study among health workers who are involved in the delivery of HIV counselling and testing services for children and their caretakers in the Kampala and Kabarole districts of Uganda. The study participants included medical doctors, nurses, counsellors, social workers and administrators of 10 healthcare facilities.

In Kampala District, the study covered seven sites: Mulago National Referral and Teaching Hospital, four faith-based hospitals (Lubaga, Nsambya, Mengo and Kibuli), the AIDS Information Centre (AIC), and the Kamwokya Christian Caring Community (KCCC). AIC and KCCC are non-governmental organizations providing HIV counselling and testing services in Kampala City. In Kabarole District, study sites were: one regional hospital (Buhinga) and two faith-based hospitals (Virika and Kabarole), which are all located within Fort-Portal Municipality.

### Data collection methods

We collected data between November 2004 and April 2005, using semi-structured individual interviews, focus group discussions and key informant interviews with medical doctors, nurses, counsellors, social workers and administrators of the study institutions.

### Individual interviews with health workers

Following informed consent, a semi-structured interview guide [11] was administered to health workers who are involved in counselling and testing of HIV-infected children.

The semi-structured interview guide consisted of structured close-ended questions, which were followed by a set of open-ended, qualitative questions. Close-ended questions captured information about the respondents' demographic characteristics and training in counselling and paediatric HIV care. The open-ended questions captured information relating to the content of the training and the challenges that the service providers encounter in counselling HIV-infected children.

The three authors conducted the interviews. Each author worked with two research assistants (university graduates), who helped in organizing appointments for the interviews and also took detailed interview notes. The interviews, which each lasted 45 to 60 minutes, were conducted in English and were not audio recorded. At the end of each interview, the researcher met with the research assistants to compile a detailed write-up and to plan for the subsequent interviews.

### Key informant interviews

Administrators and heads of the participating institutions and paediatric HIV units, as well as heads of PMTCT programmes, were selected as key informants. One of the authors (JR), who is conversant with qualitative methods of investigation, conducted the interviews with the assistance of one of the co-investigators. An open-ended interview guide was used to collect data on the structural issues that affect the delivery of paediatric HIV services and the challenges of counselling HIV-infected children.

### Focus group discussions

Three focus group discussions were conducted using a discussion guide; one was held in Kabarole District (at Buhinga Hospital) and two in Kampala District (at Mulago Hospital and Kamwokya Christian Caring Community). Eligible participants (nurses, counsellors and social workers) who did not participate in individual interviews were selected for the focus group discussions (FGDs). Each FGD comprised six participants (female and male in a ratio of 2:1). The first author (JR) moderated the FGDs while one research assistant, who had experience in conducting social research, took detailed notes. The discussions were conducted in English, and were tape recorded.

### Sampling issues

We included all public and faith-based hospitals in the two districts, as well as two non-governmental organizations (AIC and KCCC) that were providing HIV services. The AIDS Information Centre was included because it was a pioneer agency for HIV counselling and testing. Although KCCC was not one of the original selected sites, health workers at Mulago and Nsambya hospitals informed us that sometimes, they received HIV-positive children who had been tested and referred from KCCC for ART and other kinds of management. At the facility level, health workers were purposively selected depending on whether they were involved in the care of HIV-infected children.

### Data analysis

Responses to open-ended questions from individual interviews were coded and entered in EpiData. Frequency tables were generated using the SPSS statistical package (version 11.5) to reflect the training, experiences and challenges involved in counselling HIV-infected children. Qualitative data were analyzed using the content thematic approach, which was guided by the Graneheim and Lundman 2004 framework [12]. We identified study themes and sub-themes following multiple reading of interview and discussion transcripts. The major theme was the challenges faced by healthcare providers in providing HIV counselling services to children. The emerging sub-themes were: child-, caretaker-, health worker- and institutional-related challenges.

We used these themes and sub-themes to code data from interview and discussion scripts. We also conducted sub-group analysis, which involved examining the themes and sub-themes in relation to each health facility in order to identify the unique and cross-cutting challenges that exist in the delivery of HIV counselling services to children. We identified verbatim quotations that were pertinent to the study themes, which we have used in the presentation of findings.

### Ethical considerations

Ethical clearance to conduct the study was obtained from the Uganda National Council for Science and Technology, and the Kampala and Kabarole district administrations, as well as from the management of the study institutions. Written informed consent to participate in the study was obtained from all the study participants.

## Results

The results presented here were obtained from interviews that were held with health workers about the challenges they face in the delivery of paediatric HIV services. The results do not include information from interviews with children and caregivers. Four of the 10 institutions involved in the study (Mulago, Nsambya, Kibuli and Buhinga hospitals) had fully fledged HIV counselling, testing and care services for children, including the provision of antiretrovirals (ARVs). The other sites provided services mainly for adults. The paediatric HIV services included counselling, testing and referral to other centres.

### Social demographic characteristics

We interviewed 60 health workers who were involved in routine provision of HIV counselling and testing for children and child caregivers. Of the 60 service providers, 40 (66.7%) were female. The majority (42 of 60; 70%) were below 40 years of age. Counsellors constituted 21 of the 60 (35%) respondents (see table 1). The number of health workers interviewed per study site ranged from four to eight.

**Table 1:** Demographic characteristics of health workers involved in HIV counselling and testing of children in Kampala and Kabarole districts

Characteristic	Frequency (n = 60)	Percentage
**Sex**		
Male	20	33.3
Female	40	66.7
**Age in completed years**		
20-29	22	36.7
30-39	20	33.3
40-49	11	18.3
50-59	6	10.0
60-69	1	1.7
**Title/current position**		
Doctors	15	25.0
Clinical officers	3	5.0
Counsellors	21	35.0
Nurse or midwife or both	12	20.0
Laboratory technician/technologists	4	6.7
Social workers	4	6.7
Others	1	1.7

In addition, 18 administrators of the study institutions participated in key informant interviews. These included administrators and heads of paediatric HIV clinics and PMTCT programmes at the study sites.

### Training and experience in counselling and paediatric HIV/AIDS care

Thirty-eight out of the 60 respondents (63.3%) had never attended any formal training in counselling. Forty out of the 60 health workers who are involved in the provision of HIV counselling and testing (66.7%) had attended a one- to two-day sensitization workshop on paediatric HIV/AIDS. Twenty-five of these 40 (62.5%) had been exposed to basic counselling skills, while others had received training in management of paediatric HIV and communication skills, as shown in Table 2. Overall, 23 of 60 (38%) respondents had worked with an agency involved in the delivery of paediatric HIV/AIDS services prior to joining the current organization.

**Table 2:** Training and experience of health workers in counselling and paediatric HIV/AIDS care

Training and experience	Frequency (n = 60)	Percentage
Had formal training in counselling		
Yes	22	36.7
Ever worked with other agency involved in paed HIV		
Yes	23	38.3
Ever attended one-two day workshop on paediatric HIV		
Yes	40	66.7
Content covered in the workshop (out of 40)		
Counselling skills	25	62.5
Disclosure	1	2.5
Communication skills	8	20.0
Identification of paed HIV	1	2.5
Management of HIV patients	8	20.0
Management of paed HIV	13	32.5
Knowledge of ARVs	1	2.5

### Challenges in providing counselling and testing services to HIV-infected children

The challenges involved in providing counselling and testing services to HIV-infected children were grouped under: child-, caretaker-, health worker- and institutional-related challenges (Table 3).

**Table 3:** Challenges in the provision of counselling and testing services to HIV-infected children*

Difficulties	Frequency (n = 59)**	Percentage
**Institutional related**		
Few staff & heavy workload	20	33.9
Lack of testing kits and other logistical support	12	20.3
Occupational hazards (pricking self and infections)	7	11.9
Lack of prior sensitization before referral for testing	6	10.2
Poor motivation of staff	3	5.1
Lack of ARVs	2	3.4
Lack of child-friendly environment	2	3.4
		
**Caretaker related**		
Unwillingness of caretakers to disclose to child	15	25.4
Caretakers refusing children to be tested	7	11.9
Caretakers look at HIV-infected children as a burden	3	5.1
Sick and weak parents	3	5.1
Clients not sympathetic to health workers due to desperation	2	3.4
Some parents deny parenthood (stigma)	2	3.4
Lack of consistency by caregivers	2	3.4
		
**Child related**		
Children cannot express themselves easily	8	13.6
Dependency nature of children	6	10.2
Children require more time for counselling	5	8.9
Most children are needy & orphans	4	6.8
Need a lot of support to adhere to treatment	3	5.1
Children have many fears - death and abandonment	2	3.4
		
**Health worker related**		
Failure to cope with knowledge demand for HIV care	14	23.7
Lack of specialized skills in paediatric counselling & management	10	16.9
Difficult of dealing with non-parents	7	11.9
Difficult to draw blood from children	4	6.8
Difficult to disclose to children	3	5.1
Caretakers refuse other monitoring tests for ART	2	3.4

*Responses to open-ended questions posed to healthcare providers were coded into categories. Multiple responses were noted.

**One respondent did not respond to the question on challenges.

### Child-related challenges

Health workers stated that children were unable to express themselves, and depended on adults for care and support. In addition, children required more time for counselling. This was supported by additional information from the focus group discussions and key informants. The health workers stated that:

Some children are sent alone to hospital and cannot explain much. (Health worker, Mulago Hospital)

Children are emotionally moving, get attached to health workers easily and become dependent. Some children refuse to take drugs and require a counsellor who may not be available all the time. (Health worker, Buhinga Hospital)

Children have many questions which need to be answered and this takes a lot of time yet clients are too many. (Health worker, Mulago Hospital)

Some children, especially adolescents, who know they are HIV positive, ask questions about sexuality, whether they will marry and have children of their own. These are difficult questions which take a lot of time and without readily available answers. (Health worker, Nsambya Hospital)

Children, unlike adults, are more delicate; they need patience and understanding which most of us lack as we are used to handling adults. (Health worker, AIC)

These findings show that health workers are constrained by time to respond to the many questions raised by children during counselling sessions. Some health workers are not well trained to handle HIV-infected children; hence the fear of attachment and emotional challenges. The health workers are more comfortable with and are used to handling adults.

Health workers observed that some of the children are needy and lack support. The study also identified that some children, due to their age, perception of illness and the fears associated with HIV/AIDS, find it difficult to adhere to medication. Health workers struggle to deal with the fears of HIV-infected children, such as the fear of death:

Some of the children have watched their parents fall sick and die, so they relate their lives to such experiences. One of the children in a counselling session asked me whether she was going to die like her mother with a lot of pain. Sometimes she would refuse to eat, cry a lot and would not explain much when asked by the grandmother. So if you have many of such children under your care, with the many numbers of patients we see, it becomes very difficult to help them adequately. You also burn out. (Health worker, Mulago)

This explanation by the health worker shows that the fears of children are compounded by their own experiences of seeing their parents or relatives die of HIV/AIDS. Findings also show that such complex scenarios strain health workers' capacities to effectively counsel children.

Fear of stigma and discrimination in society, uncertain future and the likelihood of being denied love and general care following HIV diagnosis were some of the other major fears of children, as mentioned by the health workers:

HIV infected children have many fears, like the fear of death and abandonment, once they know that they are HIV positive. These fears need to be addressed, which is too demanding for health workers. (Health worker, Nsambya Hospital)

I counselled a child who was bitter with her aunt and every one at home because they had removed him from school saying he was always sickly. His life improved with both treatment and when he was taken back to school. (Health worker, Nsambya)

### Caretaker-related challenges

Health workers are also constrained by the unwillingness of child caretakers to disclose the condition to the children (15 of 59; 25%), refusing to have children tested (seven of 59; 12%), physical weakness and sickness of carers (three of 59; 5%) and some caretakers looking at HIV-infected children as a burden:

Some parents, especially men, are unwilling to have children tested due to fear of being identified with these children. If a child tests HIV positive some people think it means even the parent is positive. (Health worker, Virika Hospital)

Most parents tend to be protective and resist disclosure. As one said, I know my child better, it's not the right time to tell him ... (Health worker, Buhinga Hospital)

Direct (biological) parents fear to disclose HIV status to their children for fear to be blamed by their children. (Health worker, AIC).

Other challenges were lack of support for HIV-infected children and their caregivers, a situation that makes them look up to health workers to meet all their needs. Caretakers of children find it difficult to visit health facilities regularly due to lack of money for transport. Stigma, denial of parenthood and lack of consistency by caretakers also emerged as major challenges:

Some caretakers discriminate against HIV-positive children. Some are removed from school; others are delayed to be taken to hospital when they fall sick because some of the caretakers think it's wastage of money since those children will die soon. (Health worker, Mulago)

Many caretakers have a negative attitude towards educating HIV-positive children compared to HIV-negative children. Although ARVs for children are now becoming more available, many people still think it is a waste of money to educate HIV-positive children who will die soon anyway. (Health worker, KCCC)

We have seen some parents who come saying they are just helping such children or they are aunties. But with time we have found some are biological parents to these children. Parents fear that their HIV status would be identified with that of their children. It becomes difficult and challenging to counsel such children when they are denied parenthood in public places, which is a pity. (Health worker, Buhinga)

Children are brought to the clinic by different people, sometimes by a mother, grandmother, sibling and neighbour. So there is no continuity in counselling and guidance provided to caretakers. As a health worker, sometimes you are not sure what each of the caretakers knows about the child's condition. (Health worker, Mulago)

### Health worker-related challenges

A quarter of the health workers (14 of 59; 24%) were constrained by inadequate knowledge about paediatric HIV care and the lack of paediatric counselling skills:

Some of us have never been trained in counselling, so sometimes you do not know what to do next. (Health worker, Buhinga)

Some of us are general health practitioners although we are helping children. We need support from those with more experience in pediatric HIV care. (Health worker, Buhinga)

Inability to provide for the general needs of HIV-infected children; For instance, we lost a 17 year old who was staying with a grandmother due to lack of proper nutritional care. This child still stands out in my mind. (Health worker, Nsambya)

Health workers find it difficult to draw blood from children for both HIV testing and monitoring tests like the CD4 count and viral load testing. The laboratory workers expressed concern that in some cases, children are sent to laboratories without prior counselling and explanation about blood draws. This, coupled with the pain suffered during the blood draw process, makes it difficult for laboratory personnel to cope with the emotional and physical stress of the affected children.

Health workers had difficulties in disclosing the HIV infection status to children due to fear of negative outcomes, such as depression and refusal to take medication. Other challenges faced by health workers were: difficulties in communicating with and counselling children; dealing with adolescents, sexually abused and sexually active HIV-infected children; and the inability to meet the general needs of children.

The issue of handling sexually active children featured more prominently in Mulago and Kabarole hospitals. Some of the children at these centres were adolescents and were more likely to be sexually active:

HIV-positive adolescents are difficult to handle, some are sexually active, with a risk of re-infection and further spread of HIV/AIDS. I am sure most health workers do not know what to do in such cases. (Health worker, Kabarole)

It is difficult to counsel HIV-infected children who have been sexually abused, especially by close relatives. (Health worker, Mulago Hospital)

We find it very difficult to counsel children who have been sexually abused. This is because many of us health workers have not been trained to address issues of sexuality. (Health worker, Mulago)

### Institutional-related challenges

Challenges under this category included the lack of or inadequate ARVs for children, the lack of a child-friendly environment at health facilities, and the lack of referral networks for paediatric HIV care. Findings from focus group discussions and key informant interviews confirmed these challenges:

ARVs for children are still limited and there is a general problem of limited ARV formulations for children. This makes counselling for adherence difficult, especially where elderly caregivers are involved. (Health worker, Mulago)

There is inadequate space at the clinic. This limits the area children have for play and interaction to facilitate comprehensive assessment of children's needs in a natural atmosphere. (Health worker, Nsambya Hospital).

We lack child-friendly services, including play area, drawings on walls ... to make children feel free. (Health worker, Kibuli Hospital)

Lack of appropriate guidelines on child counselling was also mentioned at Nsambya, Buhinga and Mulago hospitals:

The policy on testing children is not clear and health workers lack guidelines on counselling children, especially on issues of disclosure. (Health worker, Nsambya)

We also lack information and education materials like posters and reference guidelines on HIV counselling and care for children. (Health worker, Buhinga)

Other institutional challenges mentioned included: limited staff leading to heavy work load; shortage of testing kits and other logistics; lack of, or inadequate protection against occupational hazards like pricking and infections like tuberculosis; lack of comprehensive HIV/AIDS counselling; and lack of sensitization at health facilities prior sending patients to laboratories:

The major problem we face is the inadequate number of counselors. So, clients wait for long and we also get exhausted. (Health worker, Mulago Hospital)

Counselling is increasingly becoming relevant in the hospital setting but not provided for by the Ministry of Health in its structures. So, when a centre starts offering HIV counselling and testing, the existing health workers take on counselling as an added responsibility over and above their normal work. (Health worker, Buhinga Hospital).

## Discussion

In this study, we explored the challenges faced by health workers and institutions in the delivery of HIV counselling and testing services for children in Uganda. Several challenges were identified at the institutional, caretaker, child and health provider levels. The challenges could be due to the fact that HIV counselling and testing of children is relatively new in Uganda and some health facilities have not yet built capacity and experience to handle this challenging task.

One-third of the health workers had attended courses in HIV counselling, and fewer had trained in paediatric HIV/AIDS care. The majority had attained some knowledge on paediatric HIV through one- to two-day workshops.

This is not surprising: the scale up of paediatric HIV/AIDS care has been implemented in Uganda since 2005 and is still limited. Currently, national, regional and a few private, not-for-profit hospitals are providing specialized paediatric HIV/AIDS care services in Uganda. Although HIV testing and counselling services for adults extend right through to the primary health care level (Health Centre IV and III), there is still a challenge of incorporating child counselling and testing demands in the national scale up of HIV care.

The situational analysis for paediatric HIV/AIDS care in Ethiopia also indicates that the majority of the child health service providers are not trained in paediatric HIV/AIDS care and hence lack the confidence and skills to handle children [13]. Qazi *et al *also cite the limited number of trained staff in HIV and integrated management of childhood illnesses as a challenge to scaling up ART for children [4]. The professional expertise in paediatrics is in short supply in many African countries, and few African or developing world health professionals have been trained in the care and treatment of HIV-infected children [7].

Healthcare providers in our study also reported difficulties in handling HIV-positive adolescents, particularly those who are sexually active or who have been sexually abused. These findings are again not surprising given the limited number of health workers who have undergone formal training in paediatric HIV counselling and care.

The general lack of supportive guidelines, information and education materials on paediatric HIV care at health facilities further exacerbates health worker constraints. Inability of health workers to meet the varied needs of children and child caregivers was another challenge. Kaddu Mukasa and colleagues, at the 14^th ^International AIDS Conference highlighted similar difficulties in counselling HIV-positive children, including the absence of a clear national policy and guidelines [14]. The general lack of established referral networks for paediatric HIV care was another key challenge faced by the health workers. This could be a reflection of the poor referral network in the country's health system [15]. Although these issues seem to be general health system challenges, they affect the health workers' ability to deliver HIV counselling and testing services to children.

Disclosure of HIV status to children was generally perceived as a more delicate and complicated matter than it was for adults. The challenges and complexities of disclosure of HIV status to children among health workers have also been documented in South Africa [16]. Domek observes the need for clinicians to work with family members and caregivers to encourage appropriate disclosure practices, a process that should be tailored to the individual child and community [17]. As highlighted by Wiener *et al*, training and support for health workers is critical for health workers to identify child and caregiver abilities, handle the disclosure process, identify sources of support and encourage open communication between children and child caregivers [18].

As more HIV-infected children survive into their teens, disclosure of HIV/AIDS infection to children is increasingly becoming necessary in clinical care. A recent study by Ferris and colleagues among Romanian children and teens revealed that in the context of highly active antiretroviral treatment, a child's knowledge of his or her own HIV infection status is associated with delayed HIV disease progression [19]. Balasini and colleagues, in an evaluation study of a disclosure model for paedatric patients living with HIV in Puerto Rico, established that both the youth and their caregivers considered disclosure as a positive event for them and their families [20]. Additionally, Instone observes that non-disclosure over a long time can lead to severe emotional and social consequences for children, and that parents or guardians are often unaware of these consequences [21]. Despite these benefits, disclosure of HIV status to children who are infected perinatally or early in their life remains difficult and controversial for families and providers [18,22].

Health workers observed that some caretakers prefer to keep the child's HIV status private due to fear of unforeseen consequences on the child and the family. Indeed, in some cases, this fear by parents resulted in delayed HIV testing for children with resultant delays in care even when care was available. Similarly, Rwemisisi and colleagues, in a qualitative study of 10 clients of The AIDS Support Organization (TASO), note that some parents were regularly worried that their children might be infected, but preferred to wait for the emergence of symptoms before considering HIV tests for fear of the child's emotional reaction, lack of perceived benefits from knowing the HIV status [23], and stigma [18].

Parents who fear stigma and emotional distress in their children require professional support [6] on how to deal with these challenges. A study done in Thailand among caregivers of HIV-infected children revealed that fear of negative consequences for the child was a common reason for non-disclosure [24]. The same study also revealed that despite the fear, the majority of the caregivers (88.7%) agreed that they would tell the children their diagnosis in future, and half of them expressed a need for help from health workers with disclosure [24].

Indeed parental fear, health worker limitations, health facility shortages and the limited availability of paediatric HIV/AIDS care services in many settings could in part explain the persistent phenomenon of children being identified as having HIV infection only when they become ill, and the ugly reality of the majority of such children dying without a chance of getting treatment [7]. Our study findings also suggest a need to increase the availability of life-prolonging and enhancing ARVs for children to restore hope among caregivers as a motivation for early HIV testing for children [23].

Our study further revealed that health workers are confronted by caregiver inabilities, which are mainly related to poverty. Our respondents revealed that often, caretakers of HIV-positive children find it difficult to visit health facilities regularly due to lack of money for transport. Indeed, Domek argues that poverty alleviation should be part of the global response for meaningful success in ending the devastating impact of HIV/AIDS [17].

Our study also documented child-related challenges, including the fact that children have many fears and questions that may not be adequately addressed by healthcare providers due to limited training and a heavy work load. The belief among some health workers that children are more emotional than adults and hence more difficult to communicate with, particularly on sensitive issues like HIV/AIDS, was also very prominent. In addition, some of the children are sent to health facilities unaccompanied, yet they cannot express themselves adequately. However, many of these issues may be a reflection of the health workers' inadequacy in handling and caring for HIV-infected children [14], coupled with the age limitations of the children.

Our study highlights health system gaps as challenges that health workers have to deal with day by day in the delivery of HIV counselling and care for children. The main challenges mentioned in this regard are the limited number of health workers, and the lack of appropriate ART formulations for children. Human resource constraints were also highlighted in other developing countries, like Ethiopia [13].

Our study also revealed that there is limited space to provide quality and child-friendly services. Some of the study sites lacked space to provide child-friendly services, including room for play, and more often, services for adults and children were combined.

The strength of our study is that it documents constraints faced by health workers in the delivery of paediatric HIV counselling and testing services in Uganda. This is critical, especially now that PMTCT and ART programmes are being scaled up in the country.

The main limitation of our study is the lack of caregiver and child perspectives on the constraints highlighted, particularly disclosure and the barriers to HIV testing. We were not able to obtain direct suggestions on how child- and caregiver-related constraints could be addressed. However, the perspectives of health workers in our study are in agreement with other studies [16,23].

This study was mainly descriptive. We could not carry out further analysis due to the small sample size. However, our study elicited some important issues that require attention to improve the delivery of paediatric HIV counselling and testing service.

## Conclusions

The major challenges in the delivery of paediatric HIV services were found to be related to the knowledge gap in paediatric HIV care, lack of counselling skills among service providers, and health system-related constraints. Training health workers in child counselling, including issues of disclosure, sexuality and sexual abuse, and addressing the fears related to death and an uncertain future, are needed to improve paediatric HIV care. Health workers should also be trained to develop skills that build beneficial relationships with child caregivers in order to improve care services. Provision of child-friendly services, guidelines and ARV formulations for children may provide a window of hope in the improvement of HIV counselling and testing services for children.

## Competing interests

The authors declare that they have no competing interests.

## Authors' contributions

JR conceived the study, developed the protocol, and participated in data collection, analysis and writing of the manuscript. CLM participated in study design, data collection, analysis and writing of the manuscript. GN advised on study design, and participated in data collection, analysis and writing of the manuscript. All authors reviewed, revised and approved the manuscript for submission.
